# Patterns of ROS Accumulation in the Stigmas of Angiosperms and Visions into Their Multi-Functionality in Plant Reproduction

**DOI:** 10.3389/fpls.2016.01112

**Published:** 2016-08-05

**Authors:** Adoración Zafra, Juan D. Rejón, Simon J. Hiscock, Juan de Dios Alché

**Affiliations:** ^1^Plant Reproductive Biology Laboratory, Department of Biochemistry, Cell and Molecular Biology of Plants, Estación Experimental del Zaidín, Consejo Superior de Investigaciones CientíficasGranada, Spain; ^2^Department of Plant Sciences, University of OxfordOxford, UK

**Keywords:** stigma, reactive oxygen species, fluorescent probes, defense, pollen, development, self-incompatibility, signaling

## Abstract

Accumulation of reactive oxygen species (ROS) in the stigma of several plant species has been investigated. Four developmental stages (unopened flower buds, recently opened flowers, dehiscent anthers, and flowers after fertilization) were analyzed by confocal laser scanning microscopy using the ROS-specific probe DCFH_2_-DA. In all plants scrutinized, the presence of ROS in the stigmas was detected at higher levels during those developmental phases considered “receptive” to pollen interaction. In addition, these molecules were also present at early (unopened flower) or later (post-fertilization) stages, by following differential patterns depending on the different species. The biological significance of the presence ROS may differ between these stages, including defense functions, signaling and senescence. Pollen-stigma signaling is likely involved in the different mechanisms of self-incompatibility in these plants. The study also register a general decrease in the presence of ROS in the stigmas upon pollination, when NO is supposedly produced in an active manner by pollen grains. Finally, the distribution of ROS in primitive Angiosperms of the genus *Magnolia* was determined. The production of such chemical species in these plants was several orders of magnitude higher than in the remaining species evoking a massive displacement toward the defense function. This might indicate that signaling functions of ROS/NO in the stigma evolved later, as fine tune likely involved in specialized interactions like self-incompatibility.

## Introduction

The term reactive oxygen species (ROS) defines molecules derived from the metabolism of oxygen such as hydrogen peroxide or superoxide radical. In a similar way, reactive nitrogen species (RNS) includes reactive molecules derived from nitrogen metabolism, mainly the nitric oxide (NO). The presence of ROS and RNS must be balanced to maintain the correct cellular functions. When they are present in high concentrations, they may cause damage to the cell or even cell death. Hence, the role of the antioxidants is very important, in order to keep the correct balance of these species.

The study of both ROS and RNS in the Reproductive Biology of plants is an emerging discipline. These molecules are able to modulate and control the complex signaling cascades regulating the pollen–pistil interactions in Angiosperms. Several studies have been carried out in plants considered as model like *Lilium longiflorum, Arabidopsis, Petunia* and a invasive plant in the United Kingdom such as *Senecio squalidus* (see review of Traverso et al., 2013). McInnis et al. (2006a,b) explored the amounts of ROS, particularly hydrogen peroxide, in stigmas and pollen from various different angiosperms by using the ROS probes DCFH_2_-DA and TMB. They demonstrated that constitutive accumulation of ROS/H_2_O_2_ appears to be a feature of angiosperm stigmas, and discussed these results in terms of a possible role for stigmatic ROS/H_2_O_2_ and pollen-derived NO in pollen–stigma interactions and defense.

A former work by Zafra et al. (2010) was aimed to determine whether relevant ROS and RNS were present in the stigmatic surface and other reproductive tissues in the olive over different key developmental stages of the reproductive process. The olive tree is an important crop in Mediterranean countries. It is a dicotyledonous species, with some peculiarities in its reproductive organs. The presence of self-incompatible genotypes in this species has been described, as well as fertilization mainly allogamous (this means that a flower will be preferentially pollinated by pollen from a different cultivar; Mookerjee et al., 2005). The self-incompatibility system in this plant is of gametophytic type, although not well determined yet.

The main conclusions of this work were that both ROS and NO are produced in the olive reproductive organs in a stage- and tissue- specific manner, and that these chemicals may play different functions depending on these parameters. Thus, ROS and NO may foster defense functions against microbial or fungal attacks at the early flowering stages, whereas they also may determine the presence of a receptive phase in the stigma later on, or regulate pollen-pistil interaction. This work developed on olive also confirmed the emission of NO through the apertural regions of the pollen grains and the pollen tubes, the absence of these chemicals in the style or the ovary, and the decrease in the presence of ROS present in the stigma when NO was actively produced by pollen grains reaching this floral structure.

Some emerging literature has also described ROS and NO in the reproductive biology of other species like *Glycine max* (Li et al., 2012), *Corylus avellana* (Beltramo et al., 2012), *Helianthus* (Sharma and Bhatla, 2013), *Elaeocarpus hainanensis* and *Michelia alba* (Liu and Lin, 2013).

We have recently conducted analyses of ROS localization in species with different types of stigmas and systems of self-compatibility in order to reach general conclusions regarding the physiological roles of these products in plant reproduction.

## Materials and Methods

The conspicuous changes in the distribution and proportion of different ROS occurring in the reproductive tissues of the olive throughout flower development have been used as a model to compare this topic in other plants. Several stages (unopened flower buds, recently opened flowers, dehiscent anthers, and flowers after fertilization) have been studied by using DCFH_2_-DA fluorophore and confocal laser scanning microscopy. The study was carried out in species with different types of stigmas and systems of compatibility.

Dissected floral buds or complete flowers were immersed in 50 μM DCFH_2_-DA (Calbiochem) in MES (2- [N- morpholino]ethanesulfonic acid)-KCl buffer (5 μM KCl, 50 μM CaCl2, 10 mM MES, pH 6.15) for 10 min followed by a wash step in fresh buffer for 15 min, and then observed in a Nikon C1 confocal microscope using an Ar-488 laser source. Negative controls were treated with MES-KCl buffer only.

The intensity of the green fluorescence was quantified by using the Nikon EZ-C1 viewer (3.30) software. Both average and standard deviation were calculated after measurement of a minimum of nine images corresponding to three independent experiments.

## Results

**FIGURE 1:**
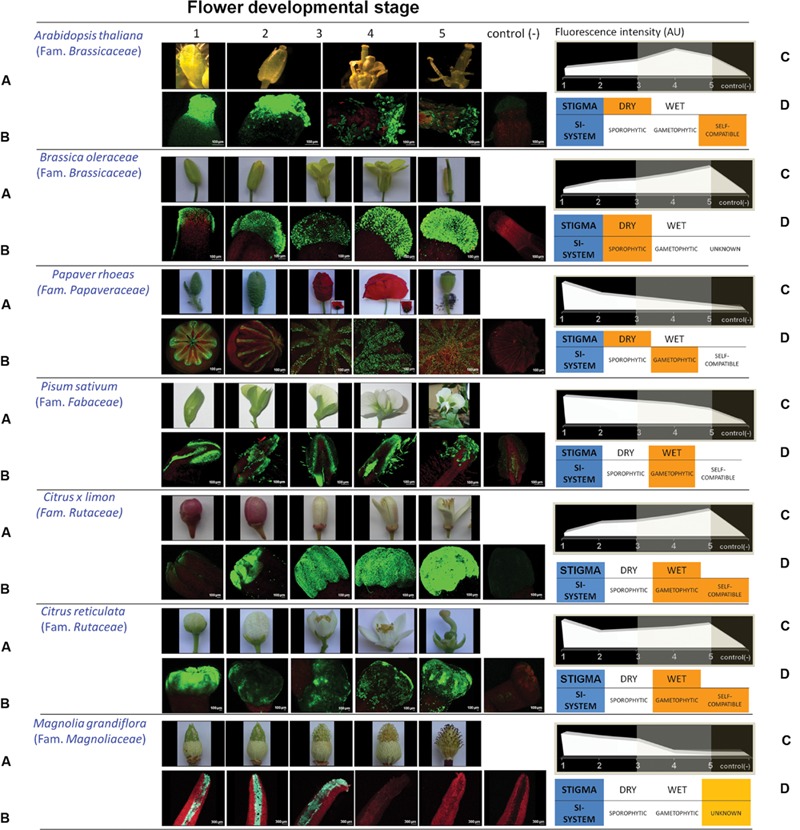
**Patterns of reactive oxygen species (ROS) distribution in different Angiosperm species. (A)** Pictures showing flower developmental stages from different Angiosperm families. Five different stages were differentiated (four stages in *Arabidopsis thaliana*, displaying dehiscent anthers before flower bud opening). **(B)** 3-D reconstructions of the whole stigma surface from multiple optical sections. The presence of ROS is shown as a fluorescent signal (green) exclusively obtained in the range of 515–560 nm emission wavelengths, whereas tissues autofluorescence was recorded in red. Green fluorescence was extensively localized in the exudate (wet stigmas), as well as in stigmatic papillae. Identical settings were used for image capture in both control/test experiments in order to ensure reproducibility. **(C)** Developmental patterns resulting from fluorescence quantification (AU: arbitrary units). **(D)** Additional details as regards to type of stigma (dry, wet) and self-incompatibility system for each plant analyzed.

Differences in the flower developmental patters of the selected species were in many cases obvious (**Figure 1A**), although a selection of similar stages was made based in the criteria described next. Flowers at stage 1 corresponded to unopened, generally green flower buds of the smaller size. Such flowers were usually dissected in order to make gynoecium available to the fluorochrome solution. At stage 2, flowers used were larger in size although still unopened; therefore removal of petals and sepals was frequently needed to make gynoecium prone to fluochrome incubation. Flowers at stage 3 were just opened and showed in most cases a significant change in the color of the petals. Gynoecia in these flowers were more easily accessible. Anthers in stage 3 contained pollen grains in most cases although anthers were not dehiscent yet. Stage 4 was characterized for anther dehiscence, with numerous pollen grains present on the stigma surface, whereas stage 5 corresponded to flowers already pollinized, displaying fallen corollas or degeneration of petals. For *Arabidopsis thaliana*, four stages were selected only, as this was the only species analyzed in which anther dehiscence took place before flower bud opening.

After treatment with DCFH_2_-DA fluorophore, low-magnification observations detected green fluorescence in all samples analyzed. This fluorescence was in all cases restricted to the stigma, and absent from the remaining parts of the gynoecium (this is the style and the ovary), which only displayed red fluorescence assigned to autofluorescence. Such autofluorescence was comparable to that one present in the negative controls where the DCFH_2_-DA fluorophore was omitted (**Figure 1B**). Anthers only showed fluorescence at dehiscence stages and during senescence. When higher magnification was used, the green fluorescence of the stigmas, resulting from DCFH_2_-DA, was observed both in the stigmatic papillae and in the stigma exudate of those species where this later was present (results not shown).

Green fluorescence was analyzed and quantified in replica of the different experiments for each one of the species analyzed and the five different developmental stages. Results of quantification are represented in **Figure 1C** by using arbitrary units of fluorescence. Different patterns of ROS accumulation were observed. In *A. thaliana, Papaver rhoeas, Pisum sativum* and *Camellia reticulata*, high levels of fluorescence were already observed at the very early flower buds (stage 1), whereas *Brassica oleracea* and *Citrus* × *lemon* displayed low levels of fluorescence at this same stage.

Stage 5, characterized by senescence of several flower organs after fecundation, also represented a differential step concerning fluorescence accumulation. In this case, high levels of fluorescence were observed in *B. oleracea, C. reticulate*, and *C* × *lemon*, whereas low level of stigmatic fluorescence appeared in the remaining species.

Relatively low fluorescence levels in comparison with the surrounding stages were observed in stages 3 and 4 for all species analyzed, coincidentally with the flower bud aperture and the putative presence of pollen grains over the stigma surface.

Reactive oxygen species detection was also performed in flowers of *Magnolia grandiflora* (Family *Magnoliaceae*), a representative species considered one of the most ancient lineages of present flowering plants. Flowers of this species are bisexual and display protogynous dichogamy in order to prevent self-incompatibility. First, female flowers open, and then a delayed second opening occurs after some time, with the flower functionally acting as a male (Losada et al., 2014 and references therein). Evidence indicates that stigma receptivity in plants of this family is brief, and has been reported to be finely coordinated with the secretion of AGPs (arabinogalactan proteins) in the stigma (Losada et al., 2014).

For this species, five developmental stages were defined as regard to female development (**Figure 1A**), including flower buds before and immediately after opening of green and white tepals (stages 1 and 2, respectively), recently opened tepals with green stigmas revealing curled tips (stage 3), colored stigmas with curled tips (stage 4), and senescent stigmas (brown) together with dehiscent anthers (stage 5).

After treatment with DCFH_2_-DA fluorophore, medium-magnification observations detected green fluorescence over the stigmas surface only, mainly at stages 1-3. Green fluorescence was absent from the anthers and other areas of the flower at the stages analyzed, which showed red autofluorescence only, comparable to that one present in the negative controls where the DCFH_2_-DA fluorophore was omitted (**Figure 1B**). Green fluorescence was analyzed and quantified. For this plant, the intensity of green fluorescence at the different replica was much higher than in the remaining species analyzed here, even after using the same experimental procedure and identical settings for image capture of the fluorescence under the same microscope equipment. Therefore, images were acquired using modified settings in order to prevent saturation of the microscope detectors. The resulting profile is displayed in **Figure 1C**, consisting in high levels of signal at the early stages (1 to 3), that quickly diminishes through stages 4 and 5 to nearly negligible levels.

## Discussion

Although a succinct number of plant species have been assayed to date, also through a limited number of developmental stages, several guidelines may arise from the present study. First, and coincidentally with the studies of McInnis et al. (2006a), Hiscock et al. (2007), Zafra et al. (2010), and Sharma and Bhatla (2013), our findings confirm that production of ROS is a prevalent feature of Angiosperm stigmas, detected in all species analyzed on this aspect so far.

A second feature demonstrated through the present and former studies consists in the limitation of the presence of detectable amounts of ROS to the tissues of the stigma surface, with the remaining floral organs lasting unlabelled (exception made to the anthers and pollen at dehiscent stages). One of the unique features of numerous stigmas in comparison with other floral organs is the presence of stigmatic exudate, extremely rich in nutrients, including sugars, lipids and proteins, which has been detected to accumulate ROS in many species (i.e., *P. sativum, Olea europaea* both *Citrus* species studied here, etc.; Serrano et al., 2008; Shakya and Bhatla, 2010; Suárez et al., 2012; Rejón et al., 2013). Moreover, we have also detected a massive presence of ROS in *M. grandiflora*, which also produces abundant secretions (Losada et al., 2014). Accumulation of ROS in the stigmatic exudate has been proposed by Hiscock et al. (2007) as a mechanism to protect against pathogen attack, on the same basis than flower nectars (Carter and Thornburg, 2000, 2004). However, and although this might represent a plausible explanation, we have detected ROS accumulation in species displaying stigmas of the dry-type like *A. thaliana, B. oleracea*, and *P. rhoeas*, therefore lacking of a significant stigma exudate. Plant species with dry and semi-dry stigmas have been described to harbor a thin pellicle which overlays the cuticle, often containing associated peroxidases (McInnis et al., 2006b). High-resolution microscopy studies would be necessary in order to assign the production of each one of the major ROS components to the tissue constituents of such stigmas.

As disclosed here, accumulation patterns for these chemicals through stigma development -a topic much less studied- offer a high level of variability among plant species. In spite of the still scarce number of stages and limited number of plants analyzed, different basic outlines have been observed. Apparently, patterns do not follow clear phylogenetic criteria, as different species from the same family do not share identical o similar models of ROS accumulation (e.g., *A. thaliana* and *B. oleracea*, both *Citrus* species studied here, and some other examples not shown -*O. europaea* and *Jasminum excelsior*-).

Sharp differences among species are visible just at the very early stages of flower development (stages 1-2), corresponding to unopened flowers. Zafra et al. (2010) discuss many of the physiological scenarios, which may concur at such stages, including the presence of high metabolic rates at the papillae and the surrounding tissues, and the defense issue mentioned above. What seems doubtful at these stages is the involvement of ROS in stigmatic receptivity and/or pollen-pistil signaling, as such stages do not physically involve pollen–pistil interaction. Then, why some plant species do not show high levels of ROS at early flowering stages? Discrepancies among species might therefore occur as the result of different rates of ROS production, for example because differences in the timing and intensity of the generated exudate, the growth rate of the floral tissues or metabolic rates (Liu and Lin, 2013).

Reactive oxygen species (mainly H_2_O_2_) scavenging has been widely correlated with launching of stigmatic receptivity, by means of the increased activity of enzymes like superoxide dismutases and peroxidases, even through the expression of new isoforms (McInnis et al., 2006b and cites therein; Sharma and Bhatla, 2013). Thus, tests for peroxidase activity have become the election method to measure pistil receptivity (Dafni and Motte Maués, 1998). Although not performed in the present study, such enzyme activities and ROS levels have been described to exhibit reverse trends during pollen–stigma interaction, the same tendency that occurs with regard to the production of NO by pollen grains reaching the stigmatic surface (McInnis et al., 2006a,b; Zafra et al., 2010; Sharma and Bhatla, 2013). In the majority of species tested here, ROS accumulation at stages 3 and 4, was overall lower than in the remaining stages. Although not particularly tested here, these observations are in good agreement with both situations: reaching of maximum stigmatic receptivity, and pistil interaction with pollen grains likely emitting NO.

Although the fine involvement of ROS in self-incompatibility mechanisms (particularly through the induction of PCD) is beginning to be undercover (Thomas and Franklin-Tong, 2004; Goldraij et al., 2006; Gao et al., 2010; Serrano et al., 2010, 2012; Allen et al., 2011; Wang and Zhang, 2011), no clear relationships between the differential patterns of ROS accumulation in the stigmas and the self-incompatibility mechanism applying for each species have been detected either. As an example, *B. oleracea* and *Citrus* × *lemon* display quite similar patterns of ROS accumulation, in spite of concealing different self-incompatibility systems (**Figure 1D**). On the contrary, species with similar self-incompatibility systems may differ broadly in their ROS-accumulating profiles (i.e., both *Citrus* species analyzed here, *P. rhoeas* and additional species not shown). Moreover, *A. thaliana* (self-compatible) and *P. rhoeas* (gametophytic SI) share rather similar profiles. Finally, *M. grandiflora* shows a nearly unique ROS-accumulating pattern. The prevalence of this type of profiles among other ancient species (either with protogynous dichogamy in order to prevent self-incompatibility, or other considered more evolved systems) is yet to be determined. The great contrast between the huge presence of ROS at the early stages and the near absence of these chemicals in *M. grandiflora* stigmas at those stages with pollen–stigma interactions might suggest that ROS function in these stigmas could be strongly unbalanced toward the defense function. Finely tuned signaling interactions among pollen and stigma, might be then reduced or absent in this primigenius and singular plant, and appear later in evolution. However, these premises have to be further assessed.

An additional topic to be comprehensively examined is flower senescence, particularly stigma senescence as per stage 5 in the present study. Programmed cell death at this stage, triggered by the rise in ROS production, although frequent and widely described for petals (Rogers, 2012), shouldn’t be considered a fully general trend in stigmas. Alternative patterns, with low production of ROS at stage 5 have been detected here in *Papaver* and *Magnolia*, again depending on the species analyzed. Finally, the role of ROS in the cellular events underlying in the gynoecium, like self-incompatibility events, pollen tube growth and directionability, and fertilization are beginning to be undercover (Heydlauff and Groß-Hardt, 2014).

## Conclusion

**FIGURE 2:**
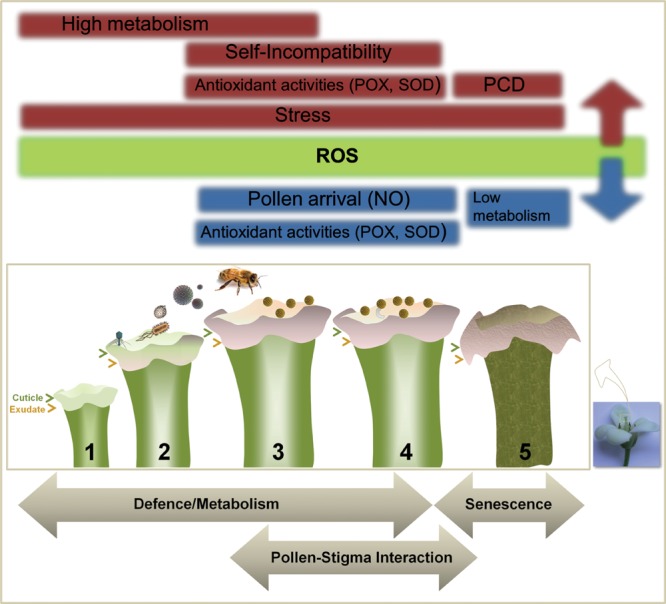
**Factors affecting ROS accumulation in Angiosperm stigma.** Numerous intrinsic and extrinsic factors may increase (red) or reduce/scavenge (blue) basal levels of ROS produced through metabolism and present mainly on the stigma surface (green), modulating their accumulation and generating different profiles highly depending on the plant species. These factors can be grouped into three major categories: defense, pollen–pistil interaction and senescence.

The multifunctional nature of ROS, generated as a consequence of metabolism, involved in numerous stress, defense and signaling functions, and modulated through numerous enzymatic and non-enzymatic systems makes their presence a valuable marker of plant (flower) physiology. The presence of ROS in pollen and stigma (**Figure 2**) is likely influenced by a number of intrinsic (histochemical composition of the stigma, presence of exudate, cuticle, differential timing of floral development for each species, self-incompatibility mechanisms) and probably also extrinsic factors such as model of pollen dispersion, pollen viability, and stress (Zinn et al., 2010). The developmental changes observed involve many biochemical systems and molecular mechanisms, which both promote and counteract the increase of ROS (Cavaiuolo et al., 2013). This should be further analyzed in the different models for reproductive biology by means of the numerous tools available in order to obtain solid evidence supporting the hypotheses displayed here.

## Author Contributions

JA and SH planned the experimental approaches. AZ and JR collected the material and carried out the experimental work. AZ, JR, and JA performed imaging experiments. All authors contributed to the preparation, reviewed and approved the manuscript.

## Conflict of Interest Statement

The authors declare that the research was conducted in the absence of any commercial or financial relationships that could be construed as a potential conflict of interest.

## References

[B1] Allen A. M. Thorogood C. J. Hegarty M. J. Lexer C. Hiscock S. J. (2011). Pollen–pistil interactions and self-incompatibility in the Asteraceae: new insights from studies of *Senecio Squalidus* (Oxford ragwort). *Ann. Bot.* 108 687–698. 10.1093/aob/mcr147 21752792 PMC3170154

[B2] Beltramo C. Torello Marinoni D. Perrone I. Botta R. (2012). Isolation of a gene encoding for a class III peroxidase in female flower of *Corylus avellana* L. *Mol. Biol. Rep.* 39 4997–5008. 10.1007/s11033-011-1296-y 22362313

[B3] Carter C. Thornburg R. W. (2000). Tobacco nectarin I. Purification and characterization as a germin-like, manganese superoxide dismutase implicated in the defense of floral reproductive tissues. *J. Biol. Chem.* 275 36726–36733. 10.1074/jbc.M006461200 10952990

[B4] Carter C. Thornburg R. W. (2004). Is the nectar redox cycle a floral defense against microbial attack? *Trends Plant Sci.* 9 320–324. 10.1016/j.tplants.2004.05.008 15231276

[B5] Cavaiuolo M. Cocetta G. Ferrante A. (2013). The antioxidants changes in ornamental flowers during development and Senescence. *Antioxidants* 2 132–155. 10.3390/antiox2030132 26784342 PMC4665434

[B6] Dafni A. Motte Maués M. (1998). A rapid and simple procedure to determine stigma receptivity. *Sex. Plant Reprod.* 11 177–180. 10.1007/s004970050138

[B7] Gao X. Zhu D. Zhang X. (2010). Stigma factors regulating self-compatible pollination. *Front. Biol.* 5 156–163. 10.1007/s11515-010-0024-7

[B8] Goldraij A. Kondo K. Lee C. B. Hancock C. N. Sivaguru M. Vazquez-santana S. (2006). Compartmentalization of S-RNase and HT-B degradation in self-incompatible *Nicotiana*. *Nature* 439 805–810. 10.1038/nature04491 16482149

[B9] Heydlauff J. Groß-Hardt R. (2014). Love is a battlefield: programmed cell death during fertilization. *J. Exp. Bot.* 65 1323–1330. 10.1093/jxb/eru030 24567492

[B10] Hiscock S. J. Bright J. McInnis S. M. Desikan R. Hancock J. T. (2007). Signaling on the stigma: potential new roles for ROS and NO in Plant Cell Signaling. *Plant Signal. Behav.* 2 23–24. 10.4161/psb.2.1.3644 19704802 PMC2633892

[B11] Li M. Sha A. Zhou X. (2012). Comparative proteomic analyses reveal the changes of metabolic features in soybean (*Glycine max*) pistils upon pollination. *Sex. Plant Reprod.* 25 281–291. 10.1007/s00497-012-0197-0 22968406

[B12] Liu N. Lin Z. (2013). Reactive oxygen species and alternative respiration in the developing flowers of two subtropical woody plants. *J. Plant Growth Regul.* 32 83–91. 10.1007/s00344-012-9278-4

[B13] Losada J. M. Hrrero M. Hormaza J. I. Friedman W. E. (2014). Arabinogalactan proteins mark stigmatic receptiviy in the protogynous flowers of *Magnolia virginiana* (Magnoliaceae). *Am. J. Bot.* 101 1–13. 10.3732/ajb.1400280 25366861

[B14] McInnis S. M. Desikan R. Hancock J. T. Hiscock S. J. (2006a). Production of reactive oxygen species and reactive nitrogen species by angiosperm stigmas and pollen: potential signalling crosstalk? *New Phytol*. 172 221–228. 10.1111/j.1469-8137.2006.01875.x 16995910

[B15] McInnis S. M. Emery D. C. Porter R. Desikan R. Hancock J. T. Hiscock S. J. (2006b). The role of stigma peroxidases in flowering plants: insights from further characterization of a stigma-specific peroxidase (SSP) from *Senecio Squalidus* (Asteraceae). *J. Exp. Bot.* 57 1835–1846. 10.1093/jxb/erj182 16698818

[B16] Mookerjee S. Guerin J. Collins G. Ford C. Sedgley M. (2005). Paternity analysis using microsatellite markers to identify pollen donors in an olive grove. *Theor. Appl. Genet.* 111 1174–1182. 10.1007/s00122-005-0049-5 16133312

[B17] Rejón J. D. Delalande F. Schaeffer-reiss C. Carapito C. Zienkiewicz K. Alché J. D. D. (2013). Proteomics profiling reveals novel proteins and functions of the plant stigma exudate. *J. Exp. Bot.* 64 5695–5705. 10.1093/jxb/ert345 24151302 PMC3871823

[B18] Rogers H. J. (2012). Is there an important role for reactive oxygen species and redox regulation during floral senescence? *Plant Cell Environ.* 35 217–233. 10.1111/j.1365-3040.2011.02373.x 21635270

[B19] Serrano I. Irene S. Pelliccione S. Salvatore P. Olmedilla A. Adela O. (2010). Programmed-cell-death hallmarks in incompatible pollen and papillar stigma cells of *Olea europaea* L. under free pollination. *Plant Cell Rep.* 29 561–72. 10.1007/s00299-010-0845-5 20352230

[B20] Serrano I. Romero-Puertas M. C. Rodríguez-Serrano M. Sandalio L. M. Olmedilla A. (2012). Peroxynitrite mediates programmed cell death both in papillar cells and in self-incompatible pollen in the olive (*Olea europaea* L.). *J. Exp. Bot.* 63 1479–1493. 10.1093/jxb/err392 22140239 PMC3276107

[B21] Serrano I. Suárez C. Olmedilla a. Rapoport H. F. Rodríguez-García M. I. (2008). Structural organization and cytochemical features of the pistil in Olive (*Olea europaea* L.) cv. Picual at anthesis. *Sex. Plant Reprod.* 21 99–111. 10.1007/s00497-008-0075-y

[B22] Shakya R. Bhatla S. C. (2010). A comparative analysis of the distribution and composition of lipidic constituents and associated enzymes in pollen and stigma of sunflower. *Sex. Plant Reprod.* 23 163–172. 10.1007/s00497-009-0125-0 20490969

[B23] Sharma B. Bhatla S. C. (2013). Accumulation and scavenging of reactive oxygen species and nitric oxide correlate with stigma maturation and pollen–stigma interaction in sunflower. *Acta Physiol. Plant.* 35 2777–2787. 10.1007/s11738-013-1310-1

[B24] Suárez C. Castro A. J. Rapoport H. F. Rodríguez-García M. I. (2012). Morphological, histological and ultrastructural changes in the olive pistil during flowering. *Sex. Plant Reprod.* 25 133–146. 10.1007/s00497-012-0186-3 22476326

[B25] Thomas S. G. Franklin-Tong V. E. (2004). Self-incompatibility triggers programmed cell death in Papaver pollen. *Nature* 429 305–309. 10.1105/tpc.016154 15152254

[B26] Traverso J. a. Pulido A. Rodríguez-García M. I. Alché J. D. (2013). Thiol-based redox regulation in sexual plant reproduction: new insights and perspectives. *Front. Plant Sci.* 4:465 10.3389/fpls.2013.00465 PMC3827552 24294217

[B27] Wang C. Zhang S. (2011). A cascade signal pathway occurs in self-incompatibility of *Pyrus pyrifolia*. *Plant Signal. Behav.* 6 420–421. 10.4161/psb.6.3.14386 21673508 PMC3142427

[B28] Zafra A. Rodríguez-García M. I. Alche J. D. (2010). Cellular localization of ROS and NO in olive reproductive tissues during flower development. *BMC Plant Biol.* 19 1–14. 10.1186/1471-2229-10-36 PMC2838403 20181244

[B29] Zinn K. E. Tunc-Ozdemir M. Harper J. F. (2010). Temperature stress and plant sexual reproduction: uncovering the weakest links. *J. Exp. Bot.* 61 1959–1968. 10.1093/jxb/erq053 20351019 PMC2917059

